# Synthesis and Characterization of Novel Succinyl Chitosan-Dexamethasone Conjugates for Potential Intravitreal Dexamethasone Delivery

**DOI:** 10.3390/ijms222010960

**Published:** 2021-10-11

**Authors:** Natallia V. Dubashynskaya, Anton N. Bokatyi, Alexey S. Golovkin, Igor V. Kudryavtsev, Maria K. Serebryakova, Andrey S. Trulioff, Yaroslav A. Dubrovskii, Yury A. Skorik

**Affiliations:** 1 Institute of Macromolecular Compounds of the Russian Academy of Sciences, Bolshoi VO 31, 199004 St. Petersburg, Russia; dubashinskaya@gmail.com (N.V.D.); qwezakura@yandex.ru (A.N.B.); 2 Almazov National Medical Research Centre, Akkuratova 2, 197341 St. Petersburg, Russia; golovkin_a@mail.ru (A.S.G.); dubrovskiy.ya@gmail.com (Y.A.D.); 3 Institute of Experimental Medicine, Akademika Pavlova 12, 197376 St. Petersburg, Russia; igorek1981@yandex.ru (I.V.K.); m-serebryakova@yandex.ru (M.K.S.); trulioff@gmail.com (A.S.T.); 4 Research and Training Center of Molecular and Cellular Technologies, St. Petersburg State Chemical Pharmaceutical University, Prof. Popova 14, 197376 St. Petersburg, Russia

**Keywords:** dexamethasone, succinyl chitosan, intravitreal delivery systems, anti-inflammatory activity

## Abstract

The development of intravitreal glucocorticoid delivery systems is a current global challenge for the treatment of inflammatory diseases of the posterior segment of the eye. The main advantages of these systems are that they can overcome anatomical and physiological ophthalmic barriers and increase local bioavailability while prolonging and controlling drug release over several months to improve the safety and effectiveness of glucocorticoid therapy. One approach to the development of optimal delivery systems for intravitreal injections is the conjugation of low-molecular-weight drugs with natural polymers to prevent their rapid elimination and provide targeted and controlled release. This study focuses on the development of a procedure for a two-step synthesis of dexamethasone (DEX) conjugates based on the natural polysaccharide chitosan (CS). We first used carbodiimide chemistry to conjugate DEX to CS via a succinyl linker, and we then modified the obtained systems with succinic anhydride to impart a negative ζ-potential to the polymer particle surface. The resulting polysaccharide carriers had a degree of substitution with DEX moieties of 2–4%, a DEX content of 50–85 μg/mg, and a degree of succinylation of 64–68%. The size of the obtained particles was 400–1100 nm, and the ζ-potential was −30 to −33 mV. In vitro release studies at pH 7.4 showed slow hydrolysis of the amide and ester bonds in the synthesized systems, with a total release of 8–10% for both DEX and succinyl dexamethasone (SucDEX) after 1 month. The developed conjugates showed a significant anti-inflammatory effect in TNFα-induced and LPS-induced inflammation models, suppressing CD54 expression in THP-1 cells by 2- and 4-fold, respectively. Thus, these novel succinyl chitosan-dexamethasone (SucCS-DEX) conjugates are promising ophthalmic carriers for intravitreal delivery.

## 1. Introduction

Inflammatory diseases of the posterior segment of the eye such as diabetic retinopathy, glaucoma, age-related macular degeneration, macular edema, and uveitis are serious medical challenges. These diseases affect the retina and choroid, resulting in visual impairment and blindness in millions of patients globally [1,2]. However, delivery of ocular drugs to the posterior segment of the eye is difficult due to the presence of anatomical and physiological ophthalmic barriers, including nasolacrimal drainage and the corneal barrier, and because of the non-target absorption of drugs by the conjunctiva [1,3,4,5,6]. Therefore, traditional dosage forms for both topical (eye drops) and systemic administration (enteral and parenteral drugs) are ineffective for the treatment of retinal diseases [7,8].

Intravitreal injections enable the delivery of anti-inflammatory agents specifically to the target sites of the posterior segment of the eye, but this medical procedure is invasive, and frequent injections can have severe side effects, including infections and retinal detachment [9,10]. For this reason, an intravitreal dosage form must have a prolonged release profile of active pharmaceutical substances over several months while still maintaining the drug concentration at an adequate therapeutic level [8,11,12]. Current intravitreal drugs include implants (e.g., FDA approved Ozurdex [13]) and various types of polymeric nanoparticles, but many of these implants and nanoparticles consist of non-degradable polymers, and this limits their wide clinical application for controlled drug release [2,3,14,15,16,17]. The use of various nanoparticles for retinal delivery does not ensure the desired months-long release profile and can also lead to increased intraocular pressure and visual impairment [11]. In addition, the physical size of both implants and particles can preclude targeted delivery specifically to the retinal cells [7]. 

One approach to the development of optimal intravitreal delivery systems is the conjugation of low-molecular-weight active pharmaceutical substances with natural polymers [1]. The diffusion rate of a drug in the vitreous humor and its elimination from the eye depend on the size of the molecule [18]. Thus, the pharmacological effects of drugs injected into the vitreous humor can be prolonged by increasing their molecular size [19], as this prevents the rapid elimination seen with low-molecular-weight components while also ensuring a targeted and controlled drug release. In addition to size, the particle surface charge is a crucial factor for successful intravitreal delivery [20,21,22]. 

The vitreous humor consists of a negatively charged three-dimensional matrix based on collagen and hyaluronic acid [14,23]. This structure allows the free movement of negatively charged and neutral particles in the vitreous humor, while the mobility of positively charged particles is strongly limited by their interactions with the anionic components of the vitreous gel [24,25,26]. Covalent conjugation with macromolecular compounds (especially non-toxic, biocompatible, and biodegradable natural polymers) can increase the size of a drug molecule, thereby increasing its residence time in the vitreous humor [27,28]. Altiok et al. [29,30] conjugated an anti-vascular endothelial growth factor (anti-VEGF) drug (sFlt-1) with polyanionic hyaluronic acid to decrease sFlt-1 clearance and increase drug retention time in the vitreous. This resulted in a tenfold increase in the drug half-life with no change in pharmacological activity. Famili et al. [31] developed hyaluronic acid-fragment antigen-binding (Fab) bioconjugates for anti-VEGF therapies. They found that conjugation of Fab with negatively charged hyaluronic acid significantly slowed in vivo clearance from rabbit vitreous humor after intravitreal injection. Compared with free Fab (the half-life in the vitreous humor was 2.8 days), Fab conjugated with hyaluronic acid with molecular weights of 40 kDa, 200 kDa, and 600 kDa cleared with half-lives of 7.6, 10.2, and 18.3 days, respectively. 

Unfortunately, polysaccharide-based conjugates for intravitreal delivery have rarely been used [32]. The aim of the present study was therefore to synthesize conjugates of the anti-inflammatory drug dexamethasone (DEX) with the natural polysaccharide chitosan (CS) to explore its potential application as an intravitreal delivery system. CS was chosen as the natural biopolymer because it is easily modified by amino groups to form both positively and negatively charged water-soluble derivatives [33]. CS and its derivatives have demonstrated safety and satisfying biocompatibility following intravitreal injections on in vivo models [34,35,36,37]. DEX was chosen as the test drug because it is a high-efficacy synthetic glucocorticosteroid (7 times more potent than prednisolone) and one of the most frequently used anti-inflammatory drugs for the treatment of eye disease, including inflammatory diseases of both the anterior (e.g., keratitis, blepharitis, allergic conjunctivitis, and dry eye) and posterior (e.g., choroiditis, uveitis, age-related macular degeneration, diabetic macular edema, and diabetic retinopathy) segments [38,39,40].

## 2. Results and Discussion

### 2.1. Synthesis and Characterization of the Succinyl Chitosan-Dexamethasone Conjugates (SucCS-DEX)

Conjugation of drug molecules with different polymers through linkers with different stabilities (amide, hydrazone, or ester linkages) provides prolonged release by controlled hydrolysis of the formed chemical bonds [41]. We used a succinyl linker to introduce the carboxyl function to DEX for subsequent conjugation with CS amino groups [42]. The resulting succinyl DEX (SucDEX) was characterized by ^1^H nuclear magnetic resonance (^1^H NMR) spectroscopy (Figure S1). 

**Figure 1:**
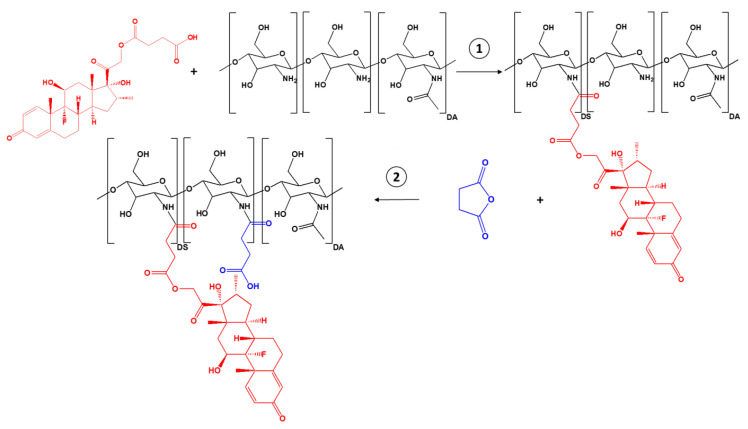
Synthesis scheme for SucCS-DEX.

**Figure 2:**
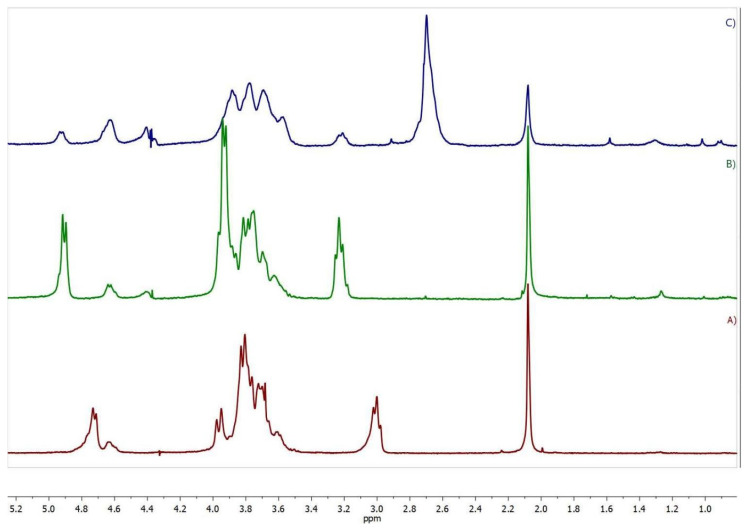
^1^H NMR spectra (400 MHz, D_2_O/trifluoroacetic acid) of (**A**) CS, (**B**) CS-DEX-10, and (**C**) SucCS-DEX-10.

SucCS-DEX was prepared by a two-step synthesis (Figure 1). SucDEX was first conjugated to CS by carbodiimide chemistry. The resulting intermediate product (CS-DEX) was isolated and characterized by Fourier transform infrared (FTIR) spectroscopy (Figure S2), ^1^H NMR spectroscopy (Figure 2), and elemental analysis (Table 1). The CS-DEX-20 sample was poorly redispersed in water, so we did not use it in further tests. The degrees of substitution with DEX moieties (DS_DEX_) of CS-DEX ranged from 2 to 4% (Table 1).

The ^1^H NMR spectrum of CS-DEX (Figure 2B) revealed all the signals of the initial CS, including the signal of the acetamide protons (2.08 ppm), H-2 of the glucosamine unit (3.23 ppm), a multiplet at 3.5–4.1 ppm (H-3–H-6 and H-2 of the N-acetylglucosamine unit), and the signals of the H-1 anomeric protons at 4.64 and 4.92 ppm. The spectrum also contained signals of the DEX protons at 0.75–3.0 ppm. The signals H-2–H-6 of the glucosamine ring were chosen as the reference signals, since no DEX protons are present in this region. The DS was calculated from the DEX proton signals at 0.85–1.6 ppm (I(DEX)_0.85–1.6_), which corresponds to 14 DEX protons using the following equation ${\mathrm{DS}}_{\mathrm{DEX}}=\frac{6\;I{\left(\mathrm{DEX}\right)}_{0.85-1.6}}{14\;I\left(H-2–H-6\right)}$. The DS determined by the NMR method was in agreement with the elemental analysis data (Table 1). 

The synthesized conjugates self-assembled in aqueous media into submicron particles with a positive ζ-potential due to the presence of the protonated CS amino groups on the surface. Therefore, the second step of conjugate synthesis was the succinylation of the positively charged particles at the amino group (Figure 1, step 2); this resulted in negatively charged final particles suitable for intravitreal administration. The negative ζ-potential prevents the particles from undergoing polyelectrolyte interactions with oppositely charged ions and thus provides the stability and mobility of the conjugates in the vitreous humor environment that consists of polyanionic hyaluronic acid [43]. Succinic anhydride (SA) was chosen as the surface modifier for CS since it is biotransformed in the body into succinic acid, a natural endogenous metabolite of the Krebs cycle, and is therefore non-toxic [44]. Succinylated CS, in addition to its high safety profile and low toxicity, is also less biodegradable than CS, so it is an excellent polymer platform for prolonged glucocorticoid delivery systems and has a release profile for the active pharmaceutical substance of several months [33,45,46]. The resulting compound was characterized by elemental analysis (Table 2) and ^1^H NMR spectroscopy (Figure 2).

The ^1^H NMR spectrum of SucCS-DEX (Figure 2C) as compared to the CS-DEX spectrum (Figure 2B) shows the appearance of a signal of methylene protons of the succinyl substituent at 2.7 ppm. The degree of succinylation (DS_Suc_) was calculated from the integral intensity of this signal (the number of protons is 4) using the following equation: ${\mathrm{DS}}_{\mathrm{Suc}}=\frac{6\;I\left({\mathrm{CH}}_{2}\right)}{4\;I\left(H-2–H-6\right)\;}\times 100\%$. DS_Suc_, determined by the NMR method, which agreed with the elemental analysis data. The DS_DEX_ of SucCS-DEX determined by the NMR method (Table 2) was in agreement with that of CS-DEX (Table 1), which indicates the absence of hydrolysis of the SucDEX substituent in the CS-DEX succinylation process. The DS_Suc_ in the SucCS-DEX was about 64–68%. The DEX content in SucCS-DEX samples was determined by UV spectroscopy (Figure S3) at 242 nm (Table 2). The spectrum of SucCS was also recorded to confirm that the polymer itself had no absorption at 242 nm.

**Figure 3:**
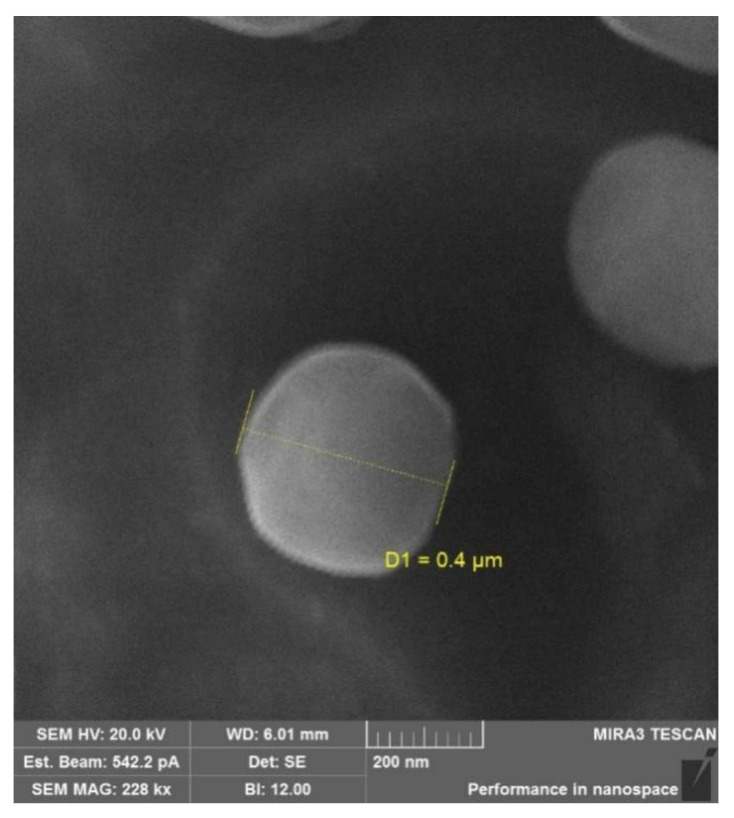
SEM images of the SucCS-DEX-10 particles.

The physicochemical characteristics (the hydrodynamic size and the ζ-potential) of the conjugates are presented in Table 3. The synthesized conjugates were capable of self-assembly in aqueous media and formed submicron-sized particles (400–1100 nm). The ζ-potential was 14–23 mV for the non-succinylated samples and −30 to −33 mV for the succinylated particles. The spherical shape of the particles was confirmed by scanning electron microscopy (SEM) (Figure 3). The average particle size on SEM images was 200–600 nm, which did not conflict with the dynamic light scattering data.

### 2.2. In Vitro DEX Release from the SucCS-DEX Conjugates

Anionic conjugates for intravitreal delivery were studied to determine the DEX release pathway (i.e., whether by amide or ester bond hydrolysis). The pharmacological activity depends on the chemical structure of the substance and the presence of substituents; therefore, we needed to determine which form of DEX (native DEX or SucDEX) is released from these polymer carriers. The mass spectrometry data showed that the hydrolysis of SucCS-DEX results in the formation of both SucDEX and DEX (the extracted ion chromatograms of the DEX forms released from SucCS-DEX-10 for 30 days are shown in Figure S4).

**Figure 4:**
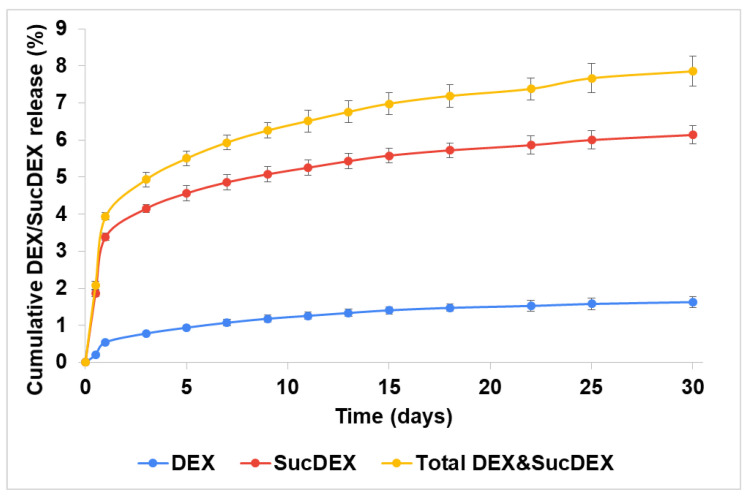
Release of DEX and SucDEX from the SucCS-DEX-5 particles; *n* = 3, error bars represent one standard deviation.

**Figure 5:**
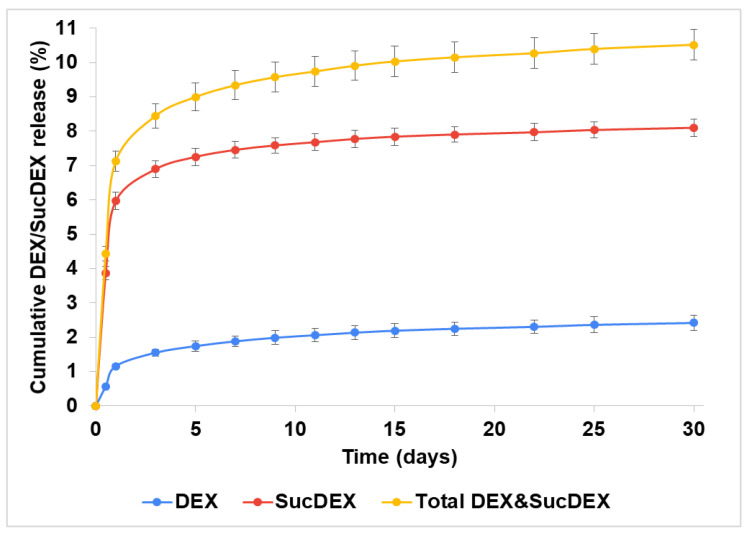
Release of DEX and SucDEX from the SucCS-DEX-10 particles; *n* = 3, error bars represent one standard deviation.

Both the DEX and SucDEX release kinetics for the SucCS-DEX-5 and SucCS-DEX-10 samples in phosphate buffered saline (PBS) at 37 °C are shown in Figure 4 and Figure 5, respectively. Under the conditions studied, the hydrolysis rate of the succinyl linker at the amide bond was higher than at the ester bond, which resulted in the favorable formation of SucDEX over DEX. 

Thus, the obtained conjugates prolonged the release of both DEX and SucDEX by a total of 8–10% for at least a month at physiological pH. Polymeric conjugates of DEX with this release profile are attractive candidates for use as intravitreal delivery systems.

### 2.3. Anti-Inflammatory Activity of the SucCS-DEX Conjugates

We needed to confirm that the two DEX forms (DEX and SucDEX) released from the SucCS-DEX conjugate retained comparative anti-inflammatory activities to that of native DEX. SucCS with DS of 65% [33] was used as a control. We first tested the influence of SucCS-DEX on in vitro THP-1 cell viability. In the absence of LPS or TNFα stimulation, all tested compounds at concentrations of 1 and 10 μg/mL significantly reduced the relative number of viable THP-1 cells (Table 4); however, no significant differences were detected between all samples under the inflammatory conditions.

We then tested SucCS-DEX-10 for its anti-inflammatory activity. We used the two standard in vitro models of THP-1 cell activation, using TNFα or LPS to induce CD54 expression. CD54 (another name is intercellular adhesion molecule-1 or ICAM-1) is a cell-surface adhesion glycoprotein that is expressed on the surface of endothelial and immune system cells, including monocytes. Proinflammatory cytokines stimulation led to augmentation of CD54 expression by human alveolar macrophages [47] and peripheral blood neutrophil [48]. THP-1 cell activation results in increased expression of CD54 on its surface.

Recombinant TNFα was added at a final concentration of 2 ng/mL and incubated for 24 h. The effect on CD54 expression was studied by flow cytometry (Table 5). We detected a reduction in the TNFα-induced expression of CD54 in THP-1 cells in the presence of SucCS-DEX-10, but expression was elevated compared to samples not induced with TNFα.

We also tested the anti-inflammatory activity of SucCS-DEX-10 in LPS-treated THP-1 cells (Table 6). We observed a significant decrease in LPS-induced CD54 expression in THP-1 cells in the presence of SucCS-DEX-10, but no differences between LPS-treated and untreated samples.

Thus, despite the minor cytotoxic effect observed for all the tested samples in THP-1 cells cultured under standard conditions, we observed a significant anti-inflammatory effect of SucCS-DEX-10. This effect was demonstrated in both infectious (LPS-induced) and sterile (TNF-induced) models of inflammation. An interesting feature was that the anti-inflammatory effect was dose-independent in both models and was the same and significant at concentrations of 1 and 10 μg/mL. SucCS-DEX-10 significantly decreased the expression of CD54 in the THP-1 cells, and this can be interpreted as an inhibition of cell activation under inflammatory conditions. This effect was detected only for SucCS-DEX-10.

## 3. Materials and Methods

### 3.1. Materials and Reagents 

In this work, we used CS from crab shells (Bioprogress, Russia) with a viscosity average molecular weight (Mη) of 37,000 and a degree of acetylation (DA) of 26% [49]. The intrinsic viscosity of CS was determined by viscometry using an Ubbelohde capillary viscometer (Design Bureau Pushchino, Russia) at 20 °C with 0.33 M acetic acid/0.3 M NaCl as the solvent. The Mη of CS was calculated using the Mark–Houwink equation: [η] = 3.41 × 10^−3^ × Mη^1.02^ [50]; [η] = 1.56 dL/g.

DEX, PBS, SA, tetrahydrofuran, 1-ethyl-3-(3-dimethylaminopropyl) carbodiimide hydrochloride (EDC), N-hydroxysuccinimide (NHS), sodium hydrogen carbonate, hydrochloric acid, trifluoroacetic acid, and deuterium oxide (D_2_O) (99.9 atom %D) were obtained from Sigma-Aldrich Co. (St. Louis, MO, USA). Pyridine was dried over sodium hydroxide and distilled before use. Other reagents and solvents were obtained from commercial sources and were used without further purification.

### 3.2. Synthesis of SucDEX

SucDEX was prepared according to the following procedure, with some modifications [42]: DEX 0.2 g (0.51 mmol) and SA 0.255 g (2.55 mmol) were dissolved in 2.2 mL pyridine. The reaction mixture was left for 7 days at room temperature, and then the product was precipitated with 30 mL 1 M HCl for purification from pyridine. The resulting white precipitate was separated by centrifugation, washed 2 times with deionized water, and dried at 60 °C for 3 h. 

### 3.3. Synthesis of the SucCS-DEX Conjugates

The CS (0.1 g, 0.53 mmol of N) was first dissolved in 5 mL 0.1 M HCl, followed by the addition of a certain amount of SucDEX dissolved in 0.5 mL tetrahydrofuran. EDC and NHS were then added to activate the carboxyl group of SucDEX, and the reaction mixture was stirred for 72 h at 20 °C (the molar ratio of the SucDEX, EDC, and NHS is presented in Table 1). The resulting product was precipitated with acetone, washed twice with acetone, filtered, dissolved in distilled water, dialyzed against distilled water for 3 days, and lyophilized. The synthesized CS-DEX (0.1 g, 0.45 mmol N) was then dispersed in 10 mL distilled water, SA was added at a 5-fold molar ratio (relative to the CS), and the reaction mixture was stirred at room temperature for approximately 7 h. Sodium hydrogen carbonate was added to the reaction mixture to bring it to pH 7–8. The resulting product was dialyzed for 3 days against distilled water and then freeze-dried.

### 3.4. Characterization of Conjugates 

The ^1^H NMR spectra were recorded using a Bruker Avance 400 instrument (Bruker, Billerica, MA, USA). Samples of SucDEX (10 mg) were dissolved in DMSO-d_6_, and the spectrum was recorded at 20 °C. Samples of CS-DEX and SucCS-DEX were prepared by dissolving 5 mg of conjugates in D_2_O. To protonate all amino groups, 5 µL trifluoroacetic acid was added to the solution. The spectra were recorded at 70 °C using a zgpr pulse sequence with suppression of residual H_2_O. 

The FTIR spectra were obtained in the attenuated total reflection mode using a Vertex-70 FTIR spectrometer (Bruker, Billerica, MA, USA) equipped with a ZnSe total reflection attachment (PIKE Technologies, Fitchburg, WI, USA). Elemental analysis (EA) was performed on a Vario EL CHN analyzer (Elementar, Langenselbold, Germany). The hydrodynamic diameter (2Rh) was measured by dynamic light scattering and the ζ-potential by electrophoretic light scattering on a Photocor Compact-Z device (Photocor, Moscow, Russia) equipped with a He-Ne laser (659.7 nm, 25 mV). The measurements were performed at a temperature of 20 °C with a scattering angle of 90°.

Particle morphology was studied by SEM using a Tescan Mira 3 instrument (Tescan, Brno, Czech Republic). Images were obtained in the secondary electron mode (SE) with an accelerating voltage of 20 kV; the distance between the sample and detector was 6 mm. The studied samples were placed on double-sided carbon tape and dried in a vacuum oven for 48 h prior to SEM observations.

### 3.5. Determination of DEX Content in the SucCS-DEX Conjugates

The DEX content in the SucCS-DEX was determined spectrophotometrically. To do this, the DEX concentration was determined in nanosuspensions (1 mg of SucCS-DEX per 20 mL double distilled water) at a wavelength of 242 nm using a calibration curve according to the equation y = 89.817x, R^2^ = 0.9923, 10 mm quartz cuvette, UV-visible spectrophotometer UV-1700 Pharma Spec (Shimadzu, Kyoto, Japan). The DEX content was then calculated per 1 mg of the SucCS-DEX.

### 3.6. In Vitro DEX Release from the SucCS-DEX Conjugates

A 1 mg sample of SucCS-DEX was dispersed in PBS (4 mL, pH 7.4) and incubated at 37 °C for 30 days. At the selected time intervals, the nanosuspension was ultracentrifuged at 4500 rpm using a Vivaspin^®^Turbo4 5000 molecular weight cut-off centrifugal concentrator and a replenishing the volume of the dissolution medium with fresh buffer. The amount and chemical structure of the released DEX or SucDEX was determined by ultra-high performance liquid chromatography—mass spectrometry. Chromatographic separation was undertaken using an Elute UHPLC (Bruker Daltonics GmbH, Bremen, Germany), equipped with a Millipore Chromolith Performance/PR-18e, C18 analytical column (100 mm × 2 mm, Merck, Darmstadt, Germany) with a Chromolith^®^ RP-18 endcapped 5-3 guard cartridges (Merck, Darmstadt, Germany), operated under a flow rate of 300 μL/min. Mobile phases were as follows: A = water: acetonitrile: formic acid—100:1:0.1 (*v*/*v*/*v*) and B = water: acetonitrile: formic acid—10:90:0.1 (*v*/*v*/*v*). Elution gradient was as follows: 0→1 min 40%B→50%B (linear gradient); 1→1.2 min 50%B→90%B (linear gradient); 1.2→2.2 min 90%B (isocratic); 2.2→2.4 min 90%B→40%B (linear gradient); 2.4→4 min 40%B (isocratic). The total run time of the method was 4 min; the injection volume was 5 μL. Mass spectra were obtained on a Maxis Impact Q-TOF mass spectrometer (Bruker Daltonics GmbH, Germany) equipped with an electrospray ionization (ESI) source (Bruker Daltonics GmbH, Germany). The instrument was operated in positive ionization mode with an electrospray voltage of 4.5 kV and a MS scanning range of 50–1000 m/z. The obtained mass spectra were analyzed using the DataAnalysis^®^ and TASQ^®^ software (Bruker Daltonics GmbH, Germany).

### 3.7. Anti-Inflammatory Activity of the SucCS-DEX Conjugates

THP-1 cells (human monocytic leukemia cells) were seeded in 50 mL plastic flasks (Sarstedt, Nümbrecht, Germany) at 37 °C in a humidified atmosphere containing 5% CO_2_, and were maintained in RPMI-1640 culture medium (Biolot, St. Petersburg, Russia) supplemented with 10% fetal bovine serum (FBS; Gibco, Thermo Fisher Scientific Inc., Bartlesville, OK, USA), 50 µg/mL gentamicin (Biolot, Russia), and 2 mM L-glutamine (Biolot, Russia), at a cell density of 0.5–1 × 10^6^ cells/mL with a medium change every 2–3 days.

For experiments, 200 µL of cell culture medium containing 1 × 10^5^ cells in suspension were seeded into 96-well flat-bottom culture plates (Sarstedt, Germany). The THP-1 cells were activated in vitro by adding 2 ng/mL TNFα (BioLegend Inc., San Diego, CA, USA) or lipopolysaccharides (LPS) from *Escherichia coli* (Sigma-Aldrich, Merck KGaA, Darmstadt, Germany) to each well (unactivated cells served as a negative control). The test compounds (SucDEX, Suc-CS, and Suc-DEX-CS-10) were added to the wells at final concentrations of 10 and 1 µg/mL (relative to DEX) and incubated with THP-1 cells for 24 h. The cells were then transferred to 75 × 12 mm flow cytometry tubes (Sarstedt, Germany) and washed with 4 mL sterile PBS (centrifugation at 300× *g* for 5 min). The washed cells were then resuspended in 50 µL fresh PBS, stained with mouse antibodies against human CD54 (Beckman Coulter Inc., Indianapolis, IN, USA) in the dark for 15 min, and washed again. Finally, the washed cells were resuspended in 100 µL fresh PBS and stained for 5 min with 250 nM PO-PRO-1 iodide (Invitrogen, Thermo Fisher Scientific, USA) and 3µM DRAQ7 (Beckman Coulter Inc., USA), as described previously [17]. At least 10,000 single THP-1 cells per each sample were acquired. Flow cytometry data were obtained with a Navios™ flow cytometer (Beckman Coulter, USA) equipped with 405, 488, and 638 nm lasers and analyzed using Navios Software v.1.2 and Kaluza™ software v.2.0 (Beckman Coulter, USA). The data were presented as the percentage of viable cells per sample ± standard error (SE), and the intensity of CD54 expression was ultimately measured as mean fluorescence intensity (MFI) on the cell surface of viable THP-1 cells.

## 4. Conclusions

We designed a two-step synthesis of SucCS-DEX conjugates for potential application as a prolonged release intravitreal delivery system. We first used carbodiimide chemistry to conjugate CS to DEX via a succinyl linker to form a hydrolysable amide bond. The resulting conjugates had a DS_DEX_ of 2–4% (DEX content of 50–85 μg/mg), a size of 450–1000 nm, and a positive ζ-potential of 14–23 mV. Next, we modified the surface of the synthesized particles with SA to obtain a negatively charged system to level out the unwanted electrostatic interaction of CS with the vitreous contents following intravitreal injection. 

As a result, we produced particles 400–1100 nm in size with a ζ-potential of −30 to −33 mV and a DS_Suc_ of 64–68%. The DEX content in the succinylated conjugates did not change compared to the initial conjugates, indicating an optimal selection of synthesis conditions and satisfactory reliability of the proposed procedure. In vitro tests showed that the developed conjugates sustained the release of the active pharmaceutical substance in the form of both DEX and SucDEX (8–10% in total for 1 month). Obviously, the vitreous environment has specific conditions (e.g., high viscosity and the presence of enzymes) that will affect the hydrolysis and release of the drug from this type of conjugate. Nevertheless, we expect that the achieved release profile will maintain therapeutic concentrations of DEX in the vitreous for several months due to the low biodegradation of succinylated CS and the sufficiently large particle size, thereby reducing side effects and the need for frequent injections. In addition, the developed conjugates demonstrated significant anti-inflammatory effects in both sterile (TNFα-induced) and infectious (LPS-induced) models of inflammation, as confirmed by 2- and 4-fold suppression, respectively, of CD54 expression in THP-1 cells. Based on the current results showing an improved release profile and the anti-inflammatory potential of the designed polymeric systems, we intend to expand this research to in vivo experiments aimed at creating an intravitreal DEX delivery system with improved pharmacological characteristics.

## Figures and Tables

**Table 1:** Synthesis conditions and characterization of CS-DEX conjugates.

Sample	Molar Ratio of Reagents				ω (%)		DS_DEX_ ** (%) by EA	DS_DEX_ (%) by NMR
	CS	EDC *	NHS *	SucDEX	C	N		
CS-DEX-5	1	0.05	0.05	0.05	40.07	6.749	1.7	1.8
CS-DEX-10	1	0.1	0.1	0.1	40.09	6.424	3.1	2.9
CS-DEX-20	1	0.2	0.2	0.2	39.27	6.145	3.8	3.9
CS	-	-	-	-	41.55	7.449	-	-

* EDC—1-ethyl-3-(3-dimethylaminopropyl) carbodiimide hydrochloride, NHS—N-hydroxysuccinimide. ** SD_DEX_ was calculated from elemental analysis data using the following formula: $\left(\frac{\omega \left(C\right)\mathrm{CS}-\mathrm{DEX}}{\omega \left(N\right)\mathrm{CS}-\mathrm{DEX}}-\frac{\omega \left(C\right)\mathrm{CS}\;}{\omega \left(N\right)\mathrm{CS}}\right)\frac{M\left(N\right)}{M\left(C\right)\;n}\times 100\%$, where ω is the mass fraction of the element in the sample, M is the molar mass of the element, *n* = 26 (the number of C atoms in SucDEX).

**Table 2:** Characterization of SucCS-DEX.

Sample	ω (%)		DS_Suc_ * (%) by EA	DS_Suc_ (%) by NMR	DS_DEX_ (%) by NMR	DEX Content (μg/mg)
	C	N				
SucCS-DEX-5	31.58	3.868	65	64	1.8	50
SucCS-DEX-10	30.40	3.575	66	68	3.0	85

* DS_Suc_ was calculated from elemental analysis data using the following formula: ${\mathrm{DS}}_{\mathrm{Suc}}=\left(\frac{\omega \left(C\right)\mathrm{SucCS}-\mathrm{DEX}}{\omega \left(N\right)\mathrm{SucCS}-\mathrm{DEX}}-\frac{\omega \left(C\right)\mathrm{CS}-\mathrm{DEX}}{\omega \left(N\right)\mathrm{CS}-\mathrm{DEX}}\right)\frac{M\left(N\right)}{M\left(C\right)\;n}\times 100\%$, where ω is the mass fraction of the element in the sample, M is the molar mass of the element, and *n* = 4 (the number of C atoms in SA).

**Table 3:** Physicochemical characteristics of CS-DEX and SucCS-DEX (mean ± standard deviation, *n* = 3).

Sample	2Rh (nm)	ζ-Potential (mV)
CS-DEX-5	816 ± 268	22.5 ± 0.5
SucCS-DEX-5	916 ± 326	−32.1 ± 0.5
CS-DEX-10	700 ± 252	14.9 ± 0.8
SucCS-DEX-10	950 ± 330	−30.9 ± 0.7

**Table 4:** The influence of DEX, SucDEX, SucCS, and SucCS-DEX-10 solutions on THP-1 cell viability under inflammatory conditions (mean ± SE).

Samples	Concentration (μg/mL)	w/o LPS or TNF	LPS (1 μg/mL)	TNFα (2 ng/mL)
Negative control (*n* = 11)	-	95.8 ± 0.3	92.40 ± 0.7	87.8 ± 1.6
DEX (*n* = 6)	1	94.0 ± 0.8 *	91.49 ± 1.2	87.1 ± 0.9
	10	92.6 ± 0.8 *	89.54 ± 1.5	85.9 ± 1.4
SucDEX (*n* = 6)	1	94.7 ± 0.5	91.94 ± 1.1	88.6 ± 0.8
	10	94.0 ± 0.6 *	88.8 ± 1.8	84.6 ± 1.1
SucCS (*n* = 6)	1	94.2 ± 0.5 *	92.9 ± 0.7	88.8 ± 1.1
	10	94.4 ± 0.6 *	92.1 ± 0.9	89.5 ± 0.9
SucCS-DEX-10 (*n* = 6)	1	94.4 ± 0.5 *	91.2 ± 0.8	87.33 ± 1.3
	10	91.8 ± 0.9 *	91.4 ± 0.7	85.23 ± 1.2

*—the differences versus the negative control samples are significant, according to the Mann–Whitney U-test with *p* < 0.05.

**Table 5:** The influence of DEX, SucDEX, SucCS, and SucCS-DEX-10 solutions on CD54 expression in THP-1 cells under TNFα-stimulated inflammatory conditions, mean ± SE.

Sample	Concentration (μg/mL)	w/o TNF	TNFα (2 ng/mL)	TNF vs. w/o TNF, *p*
Negative control (*n* = 11)	-	0.51 ± 0.02	3.3 ± 0.5	<0.001
DEX (*n* = 6)	1	0.47 ± 0.03	1.9 ± 0.2	<0.001
	10	0.55 ± 0.05	2.01 ± 0.10	<0.001
SucDEX (*n* = 6)	1	0.46 ± 0.01	2.0 ± 0.2	<0.001
	10	0.46 ± 0.02	2.1 ± 0.2	<0.001
SucCS (*n* = 6)	1	0.43 ± 0.06	2.2 ± 0.4	<0.001
	10	0.43 ± 0.06	2.4 ± 0.4	<0.001
SucCS-DEX-10 (*n* = 6)	1	0.48 ± 0.02	1.2 ± 0.3 *	0.030
	10	0.49 ± 0.02	1.04 ± 0.19 **	0.018

* and **—differences with 2 ng/mL TNF-treated control sample are significant according to the Mann–Whitney U-test with *p* = 0.004 and *p* = 0.008, respectively.

**Table 6:** The influence of DEX, SucDEX, SucCS, and SucCS-DEX-10 solutions on CD54 expression on THP-1 cells under the LPS-stimulated inflammatory conditions, mean ± SE.

Sample	Concentration (μg/mL)	w/o LPS	LPS (1 μg/mL)	LPS vs. w/o LPS, *p*
Negative control (*n* = 11)	-	0.51 ± 0.02	2.6 ± 0.9	<0.001
DEX (*n* = 6)	1	0.47 ± 0.03	2.1 ± 0.9	0.012
	10	0.55 ± 0.05	3.0 ± 1.2	<0.001
SucDEX (*n* = 6)	1	0.46 ± 0.01	1.4 ± 0.5	<0.001
	10	0.46 ± 0.02	1.8 ± 0.8	<0.001
SucCS (*n* = 6)	1	0.43 ± 0.06	1.1 ± 0.2	0.016
	10	0.43 ± 0.06	1.0 ± 0.3 *	0.039
SucCS-DEX-10, (*n* = 6)	1	0.48 ± 0.02	0.69 ± 0.16 **	0.231
	10	0.49 ± 0.02	0.54 ± 0.12 **	0.693

* and **—differences with the 1 μg/mL LPS-treated control sample are significant according to the Mann-Whitney U-test, with *p* = 0.047 and *p* = 0.016, respectively.

## Data Availability

The data are contained within the article and Supplementary Materials.

## Supplementary Materials

The following are available online at https://www.mdpi.com/article/10.3390/ijms222010960/s1.
