# Supplementary material for: Synthesis and Characterization of Novel Succinyl Chitosan-Dexamethasone Conjugates for Potential Intravitreal Dexamethasone Delivery
Source: Int J Mol Sci. 2021 Oct 11;22(20):10960. doi: 10.3390/ijms222010960 (PMC8535746; doi:10.3390/ijms222010960)
Supplement: Supplementary file 1 [file ijms-22-10960-s001.zip › ijms-1397299-supplementary.pdf]

Supplementary Materials

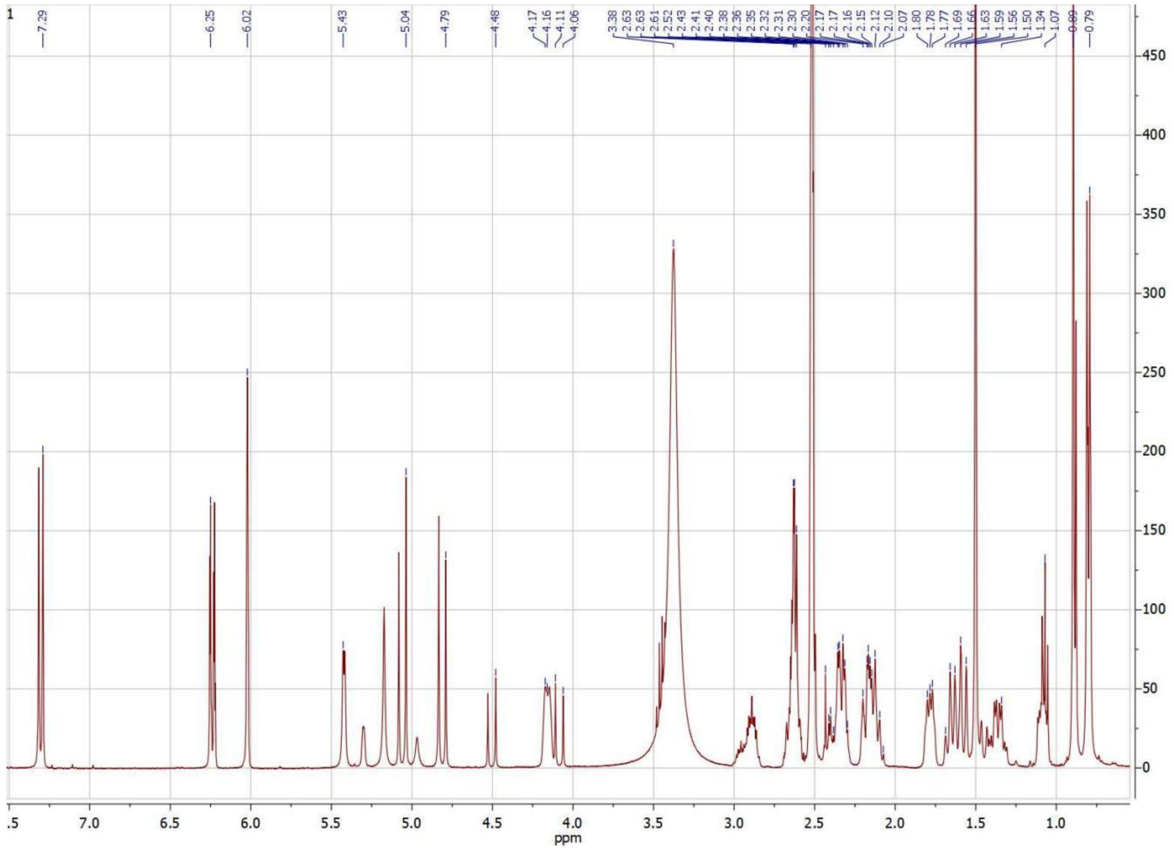

Figure S1. ^{1}H NMR (400 MHz, DMSO-d6) spectra of succinyl dexamethasone (SucDEX): δ 7.29 (d, 1H), 6.25 (dd, 1H), 6.02 (s, 1H), 5.43 (d, 1H), 5.04 (d, 1H), 4.79 (d, 1H), 4.49 (d, 1H), 4.17–4.06 (m, 1H), 3.38(s, 2H), 2.93–2.86 (m, 1H), 2.71–2.58 (m, 1H), 2.52 (s, 2H), 2.45–2.30 (m, 2H), 2.21–2.06 (m, 2H), 1.82–1.74 (m, 1H), 1.69–1.54 (m, 1H), 1.50 (s, 1H), 1.34 (dd, 1H), 1.07 (m, 1H), 0.89 (s, 3H), and 0.79 (d, 3H).

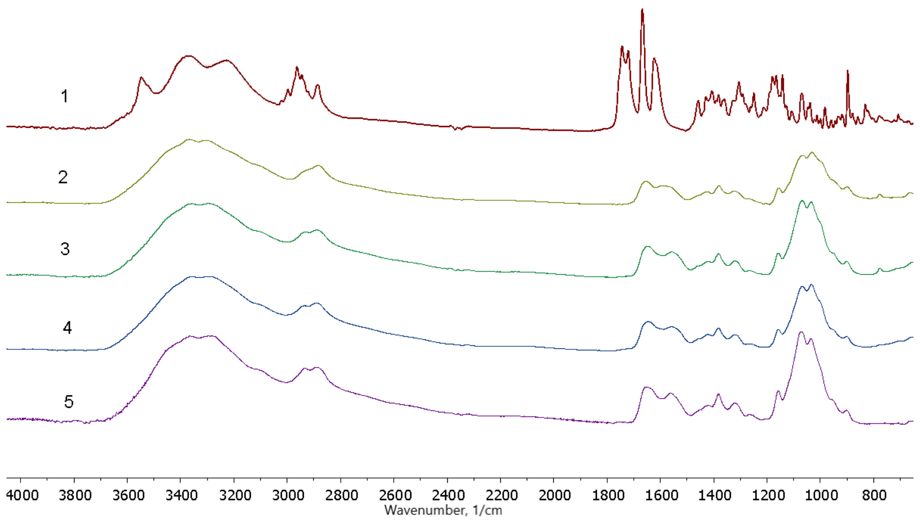

Figure S2. FTIR spectra of DEX (1), CS (2), and CS-DEX-5 (3), CS-DEX-10 (4), and CS-DEX-20 (5): the increase in the intensity of the absorption band at 2940 cm^{-1} corresponding to the vibrational transitions of the C-H DEX bonds indicates an increase in the DEX content in the conjugates.

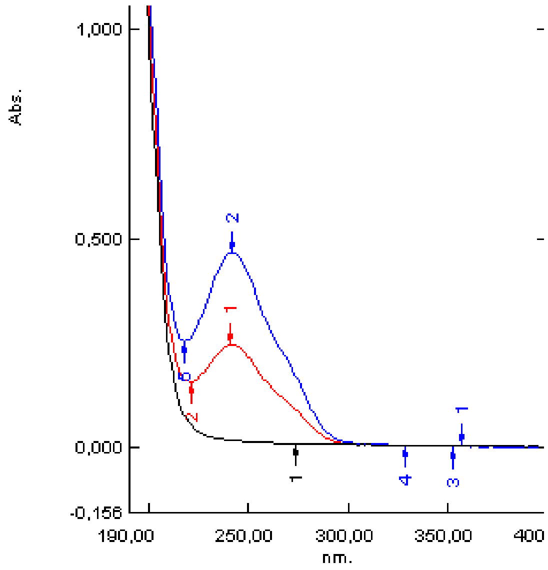

Figure S3. UV spectra of the succinyl chitosan-dexamethasone conjugates and succinyl chitosan: SucCS-DEX-5 (red), SucCS-DEX-10 (blue), and SucCS (black).

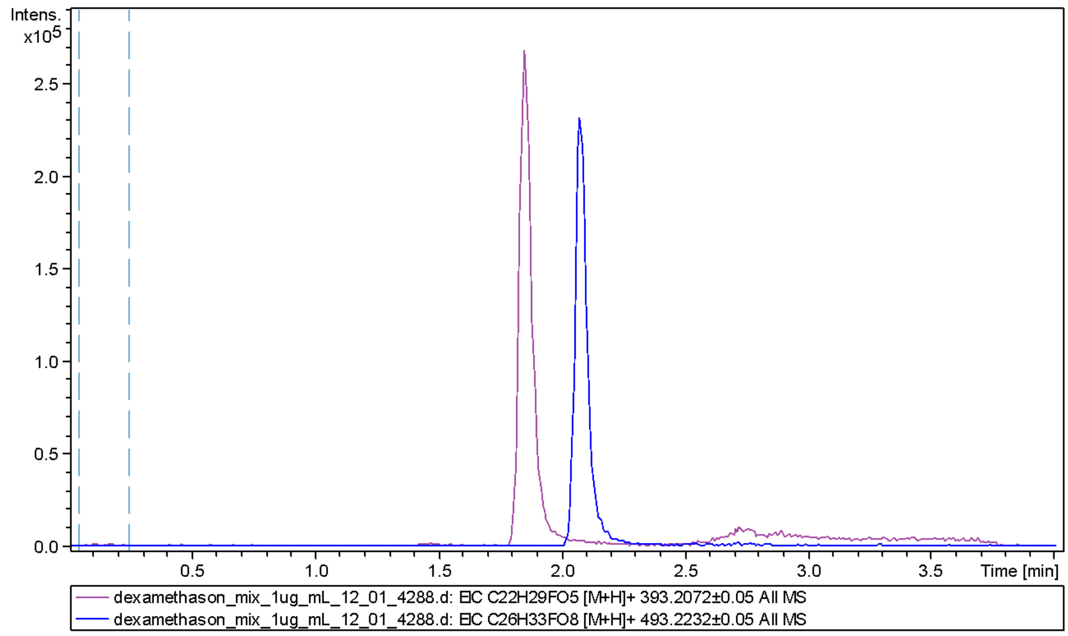

Figure S4. Extracted ion chromatograms at m/z 393.2072 (red) and m/z 493.2232 (blue) for a 1 µg/mL standard solution. m/z 393.2072 corresponds to the protonated DEX form, m/z 493.2232 corresponds to the SucDEX.
